# Editorial – Diagnostic genome sequencing in rare disorders

**DOI:** 10.1515/medgen-2023-2029

**Published:** 2023-06-13

**Authors:** Peter Krawitz, Tobias B Haack

**Affiliations:** University Bonn Institute of Genomic Statistics and Bioinformatics 53127 Bonn Germany; University of Tübingen Institute of Medical Genetics and Applied Genomics 72076 Tübingen Germany

About 8,000 rare diseases (RDs) are currently known. Although rare by definition (prevalence of 1:2,000 in the European population), they collectively constitute a major health issue, with an estimated six percent of the population being affected from one of these diseases (https://pubmed.ncbi.nlm.nih.gov/31527858/). More than 70 % of the unique RDs have a genetic cause. Despite substantial advances in diagnostics and research strategies, the diagnostic yield varies between 20–70 % depending on the disease entity and testing strategy applied. Amongst others, possible reasons for missed diagnoses include unclear disease-gene association, inaccurate models for variant prioritization, incorrect annotation and interpretation of variants, as well as technical shortcomings in bioinformatic variant detection and insufficient sequencing data. As for the last point, the broad implementation of genome sequencing (GS) is the next logical step to narrow the diagnostic gap in rare disease genetics. However, routine application of GS is challenged by laboratory, bioinformatic, interpretation, and financial issues, not to mention the need for a health system that provides the framework for GS in routine healthcare. While GS is expected to become available for selected individuals as part of the *Modellvorhaben Genomsequenzierung nach § 64e SGB V* in 2024, concerted action from scientists, medical doctors, bioinformaticians, and health care managers is needed to ensure streamlined diagnosis and data management according to FAIR standards (https://www.nature.com/articles/sdata201618). The latter is a prerequisite to fully exploit the wealth of information provided by a full genome’s sequence in the future and advance our ability to interpret unknown aetiological variation, in the coding and non-coding regions of the genome.

This issue of *Medizinische Genetik* covers aspects related to quality assurance in genome diagnostics, the value of rapid GS in critically ill children, and the role of AI-based approaches in variant classification.

The first manuscript ‘Quality assurance within the context of genome diagnostics (A German perspective)’ by Kraft,* et al.* summarizes the current state of quality assurance in genome diagnostics. With reference to national and international guidelines and recommendations, relevant topics are discussed along the organizational structure of the DIN EN ISO 15189 including legal and regulatory aspects in addition to the entire diagnostic process from preanalytics to reporting of findings. The article by Auber, *et al.* highlights the value but also the technical challenges of fast-track genomes in the clinic, which is a use case that is still underserved. The work by Lesmann, *et al.* delineates the importance of the clinical assessment of a patient by medical professionals and argues how AI will complement and not replace physicians. All examples show cased in these articles illustrate that the problem is not simply solved by transitioning from exome to genome. The challenge ahead is developing tests and operating procedures that are suitable for individual patients with a likely genetic cause.

As outlined in the *National Strategy for Genomic Medicine—genomDE*, the current developments in human genetics hold the potential to substantially improve the prevention, diagnosis and treatment of both rare and common disease.

